# Focal Parenchymal Atrophy of the Pancreas Is Frequently Observed on Pre-Diagnostic Computed Tomography in Patients with Pancreatic Cancer: A Case-Control Study

**DOI:** 10.3390/diagnostics11091693

**Published:** 2021-09-17

**Authors:** Shin Miura, Tetsuya Takikawa, Kazuhiro Kikuta, Shin Hamada, Kiyoshi Kume, Naoki Yoshida, Yu Tanaka, Ryotaro Matsumoto, Mio Ikeda, Fumiya Kataoka, Akira Sasaki, Waku Hatta, Jun Inoue, Atsushi Masamune

**Affiliations:** Division of Gastroenterology, Tohoku University Graduate School of Medicine, Sendai 980-8574, Japan; miurashin@med.tohoku.ac.jp (S.M.); t-takikawa@med.tohoku.ac.jp (T.T.); kkikuta@med.tohoku.ac.jp (K.K.); hamadas@med.tohoku.ac.jp (S.H.); kkume@med.tohoku.ac.jp (K.K.); iiiiiiiiiiiiii14@hotmail.com (N.Y.); y.tanaka1055@gmail.com (Y.T.); rmat44@gmail.com (R.M.); mio-0311@watch.ocn.ne.jp (M.I.); a9mb1039@yahoo.co.jp (F.K.); akira.ss.0911@gmail.com (A.S.); waku-style@festa.ocn.ne.jp (W.H.); jinoue@med.tohoku.ac.jp (J.I.)

**Keywords:** carcinoma in situ, early pancreatic cancer, main pancreatic duct dilatation, pancreatic ductal adenocarcinoma, pancreatic intraepithelial neoplasia

## Abstract

**Simple Summary:**

Pancreatic ductal adenocarcinoma (PDAC), which accounts for the majority of pancreatic cancers, is highly lethal, and its early diagnosis is difficult. Focal parenchymal atrophy (FPA) observed on computed tomography (CT) has been reported as a characteristic imaging finding of early PDAC without identifiable masses. However, it remains unclear whether FPA is frequently observed on pre-diagnostic CT. In this study, 76 patients with PDAC in whom CT was performed between 6 months and 3 years before PDAC diagnosis, and 76 sex- and age-matched controls without PDAC were reviewed. FPA was observed corresponding to the location of the subsequent tumor detected in 26/76 (34.2%) patients on pre-diagnostic CT, whereas it was observed in 3/76 (3.9%) controls without PDAC (*p* < 0.001). FPA was less frequently found in tumors in the pancreatic head than in those in the pancreatic body or tail. FPA may predict the subsequent diagnosis of PDAC, thus serving as an important imaging sign for the early diagnosis of PDAC.

**Abstract:**

Pancreatic ductal adenocarcinoma (PDAC) accounts for the majority of all pancreatic cancers and is highly lethal. Focal parenchymal atrophy (FPA) of the pancreas has been reported as a characteristic imaging finding of early PDAC. Here, we reviewed 76 patients with PDAC who underwent computed tomography (CT) between 6 months and 3 years before PDAC diagnosis, as well as 76 sex- and age-matched controls without PDAC on CT examinations separated by at least 5 years. FPA was observed corresponding to the location of the subsequent tumor on pre-diagnostic CT in 14/44 (31.8%) patients between 6 months and 1 year, 14/51 (27.5%) patients between 1 and 2 years, and 9/41 (22.0%) patients between 2 and 3 years before PDAC diagnosis. Overall, FPA was more frequently observed in patients with PDAC (26/76; 34.2%) on pre-diagnostic CT than that in controls (3/76; 3.9%) (*p* < 0.001). FPA was observed before the appearance of cut-off/dilatation of the main pancreatic duct, suggesting that FPA might be the earliest sign of PDAC. FPA was less frequently found in tumors in the pancreatic head (3/27; 11.1%) than in those in the body (14/30; 46.7%) or tail (9/19; 47.4%). FPA may predict the subsequent PDAC diagnosis, serving as an important imaging sign for the early diagnosis of pancreatic cancer.

## 1. Introduction

Pancreatic ductal adenocarcinoma (PDAC), which accounts for the majority of all pancreatic cancers, is one of the most lethal cancers and the fourth most common cause of cancer-related deaths in Western countries [1,2]. The American Cancer Society estimates that about 60,430 individuals (31,950 men and 28,480 women) will be diagnosed with pancreatic cancer, and about 48,220 individuals (25,270 men and 22,950 women) will die because of pancreatic cancer in 2021 [3]. In Japan, the relative 5-year overall survival rate for pancreatic cancer diagnosed between 2009 and 2011 was 8.5%, which is far below the average survival rate for all cancer types (64.1%) [4]. Patients who present with symptoms generally have advanced-stage disease. Therefore, early diagnosis in asymptomatic patients is essential to improve the prognosis of PDAC, but this remains difficult [5,6,7]. In the Pancreatic Cancer Registry in Japan [8], the 5-year survival rate of patients with stage 0 pancreatic cancer (high-grade pancreatic intraepithelial neoplasia/carcinoma in situ) was 85.8% and of those with invasive carcinoma measuring 10 mm or less was 80.4%. However, these patients accounted for only a very small portion of all the patients with PDAC; patients with stage 0 accounted for 1.7%, and those with tumors measuring 3–10 mm accounted for 0.8% [8]. In the National Cancer Institute’s Surveillance, Epidemiology, and End Results Program, the 5-year overall survival rate of patients with stage IA (maximum tumor diameter of ≤2 cm) was 83.7% in 2012, but these patients accounted for 2.5% of all patients with PDAC [9]. Several reasons contribute to difficulty in the early diagnosis of PDAC, including the lack of highly sensitive and specific biomarkers, the limited accessibility of the pancreas for biopsy, and the relative inability to define sufficiently high-risk populations that could benefit from screening [5,6,7].

In cases with very early stage PDAC, including stage 0, imaging modalities usually cannot identify the mass, and only secondary findings might provide a clue. Main pancreatic duct (MPD) abnormalities, such as localized stricture/cut-off with subsequent upstream dilatation, might be key secondary findings for the early diagnosis of PDAC [10,11,12,13]. However, MPD abnormalities are not specific to PDAC and are often observed in benign pancreatic diseases. Identification of imaging signs that are more specific to PDAC is urgently needed. Recently, several studies have shown that focal parenchymal atrophy (FPA) of the pancreas is a characteristic imaging finding revealed on CT performed in patients with early stage PDAC, including those with stage 0 PDAC [13,14,15]. Miura et al. [13] reported that FPA was observed in 7/10 (70%) patients with early stage PDAC but only in 1/10 (10%) patients with benign MPD stricture/dilatation. Yamao et al. [14] reported that FPA was identified in 11/24 (45.8%) patients with small PDAC measuring less than 10 mm but only in 2/28 (7.1%) patients with benign MPD stenosis. Nakahodo et al. [15] reported that among the 46 patients suspected of early stage PDAC, FPA was observed in 15/27 (55.6%) patients with carcinoma in situ but only in 3/19 (15.8%) patients with non-malignancy. In a multi-center study of early stage PDAC in Japan, FPA was observed in 21/50 (42.0%) patients with stage 0 PDAC and 61/146 (41.8%) patients with stage I PDAC [16]. These results suggest that FPA serves as an important imaging sign for the early diagnosis of PDAC. However, it remains unknown whether FPA is frequently observed before the diagnosis of PDAC and whether FPA can predict the subsequent diagnosis of PDAC. To clarify these issues, the present study evaluated pre-diagnostic CT scans of patients with PDAC. We here show that FPA is frequently observed on pre-diagnostic CT and serves as one of the earliest imaging signs of PDAC.

## 2. Materials and Methods

### 2.1. Study Design

This was a retrospective case-control study. This study was performed in accordance with the principles of the Declaration of Helsinki, and the protocol was approved by the Ethics Committee of Tohoku University Graduate School of Medicine (Article#: 2017-1-343; approved on 24 July 2017; 2020-1-693 approved on 6 November 2020; and 2021-1-355 approved on 21 July 2021). The requirement for informed consent was waived because of the retrospective nature of the study. Clinicopathological information of the patients was obtained from medical records.

### 2.2. Subjects

Between January 2013 and December 2020, patients with a confirmed histopathological diagnosis of PDAC at Tohoku University Hospital were reviewed. Patients with intraductal papillary mucinous neoplasm with high-grade dysplasia or PDAC derived from intraductal papillary mucinous neoplasm and recurrent PDAC were excluded. Among the subjects, patients who underwent CT examinations at our or other institutions between 6 months and 3 years before the diagnosis of PDAC (“pre-diagnostic CT”) were enrolled in this study. This time frame was selected because previous studies have shown that (1) the resectability of PDAC could be improved if the PDAC is detected at least 6 months before its clinical diagnosis, (2) progressive and increasingly frequent changes on CT occur starting 12–18 months before diagnosis, and (3) CT findings definite or suspicious for PDAC were seldom noted on CT scans obtained more than 3 years before diagnosis [17,18,19]. The Union for International Cancer Control (UICC) classification (8th edition) [20] was used to determine the disease stage. The UICC stage was determined according to the pathological stage for resected PDAC cases and the clinical stage for non-resected PDAC cases.

For each case, one sex- and age (±5 years)-matched control was selected from the patients with other diseases who underwent CT examinations (diagnostic CT) at Tohoku University Hospital between April 2011 and August 2021 that revealed no evidence of PDAC and who also underwent CT examinations at least 5 years before the diagnostic CT (pre-diagnostic CT). The chief reasons for CT examinations in the 76 controls were follow-up of the following diseases: hepatocellular carcinoma (*n =* 24), gastric cancer (*n =* 19), gallbladder stone (*n =* 6), adenomyomatosis of the gallbladder (*n =* 6), biliary cancer (*n =* 6), gallbladder polyp (*n =* 5), chronic hepatitis (*n =* 3), retroperitoneal tumor (*n =* 2), hypergastrinemia (*n =* 1), colon cancer (*n =* 1), and dilatation of bile duct (*n =* 1).

### 2.3. Evaluation of CT Scans

We focused on the presence or absence of pancreatic masses (presumably missed at the time of clinical interpretation of the pre-diagnostic CT findings), cut-off and upstream dilatation (≥3 mm) of MPD, FPA, and cysts on pre-diagnostic CT. The slice thickness was 1 mm for routine contrast-enhanced CT and 5 mm for unenhanced CT. CT scanning protocols were not consistent in all the patients because the indications for CT examination were different, CT examinations were performed for a relatively longer period, and CT examinations performed at other institutions were also included.

FPA is defined as narrowing of the focal parenchyma in comparison to both the head- and tail-side parenchyma, showing a cave-in, slim, or slit-like appearance [15]. Extensive distal pancreatic atrophy, which is often found in advanced PDAC [21], was not regarded as FPA. Due to the difficulty in its detection, we did not regard the lesion in the edge of the pancreas as FPA. This study did not define an absolute criterion for the degree of depression of the FPA because the thickness of the pancreas tends to be atrophic with age and, to the best of our knowledge, the normal size of the age-corrected pancreas has not been established yet [22].

All CT scans were independently reviewed by two reviewers who were blinded to the clinical information of the patients with PDAC, including the PDAC location in the pancreas. In the control subjects, the presence of FPA was assessed in the entire pancreas due to the absence of the target region. In cases where the imaging interpretation was considered equivocal between the two reviewers, a third party reviewed the CT scans. After individual reading, they discussed and reached a consensus on each imaging report. All the reviewers had more than 10 years of experience in the management of pancreatic diseases and are board-certified gastroenterologists of the Japanese Society of Gastroenterology and board-certified pancreatology experts of the Japan Pancreas Society.

### 2.4. Statistical Analysis

Continuous variables were presented as median (range) and compared using Wilcoxon’s rank-sum test. We used Fisher’s exact test for the comparison of proportions. Statistical analyses were performed using JMP Pro version 14 (SAS Institute Inc., Cary, NC, USA). Statistical significance was set at *p* < 0.05.

## 3. Results

### 3.1. Characteristics of Patients Who Underwent CT Examinations between 6 Months and 3 Years before PDAC Diagnosis

During the study period, 738 patients were histopathologically diagnosed with PDAC at the Tohoku University Hospital. Of them, 76 (10.3%) patients underwent CT examinations between 6 months and 3 years before PDAC diagnosis. Moreover, 43 (56.6%) patients underwent pre-diagnostic CT at Tohoku University Hospital, and the remaining 33 (43.4%) patients did at other 25 institutions. The median (range) age of the patients was 72 (49–85) years at the time of PDAC diagnosis, and 43 (56.6%) patients were men (Table 1). PDAC was located in the pancreatic head in 27 (35.5%) patients, in the body in 30 (39.5%), and in the tail in 19 (25.0%). The median mass diameter on CT was 21.5 (range 0–80.0) mm. According to the UICC classification (8th edition) [20], PDAC was stage 0 in 1 (1.3%), stage IA in 15 (19.7%), stage IB in 10 (13.2%), stage IIA in 4 (5.3%), stage IIB in 16 (21.1%), stage III in 8 (10.5%), and stage IV in 22 (28.9%) patients. Pancreatic resection was performed in 45 (59.2%) patients.

Overall, 32 (42.1%) patients underwent pre-diagnostic CT examination for follow-up of pancreatic diseases (18 with pancreatic cysts, 8 with chronic pancreatitis, 4 with post-acute pancreatitis, and 2 with autoimmune pancreatitis), while 27 (35.5%) patients underwent CT examination for follow-up of non-pancreatic malignant diseases (5 with breast cancer, 4 with lung cancer, 4 with head and neck cancer, 4 with colon cancer, 2 with malignant lymphoma, and 8 with other malignancies). Other reasons for pre-diagnostic CT were follow-up for liver diseases (*n =* 4), cardiovascular diseases (*n =* 3), respiratory diseases (*n =* 2), gastrointestinal disease (*n =* 2), and other diseases (*n =* 6).

### 3.2. FPA Was Frequently Observed on Pre-Diagnostic CT

At the time of PDAC diagnosis, CT examination (“diagnostic CT”) revealed a tumor mass in 63 (82.9%) patients and MPD cut-off/dilatation in 58 (76.3%) patients. Pancreatic cysts were observed within 2 cm of tumor location in 22 (28.9%) patients and beyond 2 cm in 28 (36.8%) patients. FPA was noted in 6 (7.9%) patients; the UICC stage in these 6 patients presenting with FPA was stage 0 in 1, stage IA in 4, and stage IIB in 1 patient.

Of the 76 enrolled patients, 44 underwent CT examinations between 6 months and 1 year before PDAC diagnosis, 51 (67.1%) patients between 1 and 2 years, and 41 (53.9%) patients between 2 and 3 years (Table 2). The number of pre-diagnostic CT examinations was 1 in 33 (43.4%) patients, 2 in 25 (32.9%) patients, and 3 or more in 18 (23.7%) patients. The proportion of the contrast-enhanced CT was not different between the periods when pre-diagnostic CT was performed (79.5% between 6 months and 1 year before PDAC diagnosis, 82.3% between 1 and 2 years, and 80.5% between 2 and 3 years; *p* = 0.96). MPD cut-off/dilatation was observed on diagnostic CT in 58/76 (76.3%) patients and on pre-diagnostic CT performed between 6 months and 1 year before in 12/44 (27.3%) patients, between 1 and 2 years in 7/51 (13.7%) patients, and between 2 and 3 years in 1/41 (2.4%) patients. The frequency of MPD cut-off/dilatation increased in a time-dependent manner as approaching the diagnosis (*p* < 0.0001).

Although they were presumably missed at the time of clinical interpretation, pancreatic masses were observed in 10/44 (22.7%) patients on pre-diagnostic CT performed between 6 months and 1 year before with a median diameter of 17.5 (range 11.0–26.0) mm. Pancreatic masses were not detected on pre-diagnostic CT performed 1 year or more before PDAC diagnosis.

FPA was detected in 14/44 (31.8%) patients on pre-diagnostic CT performed between 6 months and 1 year before PDAC diagnosis, in 14/51 (27.5%) between 1 year and 2 years, and in 9/41 (22.0%) between 2 years and 3 years (Table 3). In total, FPA was observed in 26/76 (34.2%) patients on pre-diagnostic CT. FPA detection was not restricted to our institute; FPA was observed in 11/43 (25.6%) patients who underwent pre-diagnostic CT at our institute and in 15/33 (45.5%) patients who did at other 25 institutions (*p* = 0.09). Among these 26 patients, FPA was still observed in only 5 (19.2%) patients at the time of PDAC diagnosis, and FPA was no longer obvious due to the growth of invasive tumors in the remaining 21 (80.8%) patients. FPA could be detected on pre-diagnostic CT even in patients undergoing unenhanced CT only. Among the patients who underwent unenhanced pre-diagnostic CT only, FPA was detected in unenhanced CT scans obtained between 6 months and 1 year before PDAC diagnosis in 2 patients, between 1 and 2 years in 4 patients, and between 2 and 3 years in 1 patient.

### 3.3. FPA Was Less Frequently Observed in PDAC Located in the Pancreatic Head

We compared the clinical characteristics of patients who presented with FPA (*n =* 26) and those who did not (*n =* 50) (Table 4). Clinical characteristics such as sex, age, number of pre-diagnostic CT examinations, other imaging findings, and stage at PDAC diagnosis were not different between the patients presenting with FPA and those presenting without FPA. Interestingly, the location of the primary tumor was different between these patients; FPA was detected less frequently in PDAC in the pancreatic head, compared to FPA detected in PDAC in the pancreatic body or tail (*p* = 0.0045). Among the 27 patients with PDAC located in the pancreatic head, only 3 (11.1%) presented with FPA, whereas 14/30 (46.7%) patients with PDAC in the pancreatic body and 9/19 (47.4%) patients with PDAC in the pancreatic tail presented with FPA.

### 3.4. FPA Was More Frequently Observed in Patients with PDAC Than Controls without PDAC on Pre-Diagnostic CT

To assess the performance of FPA for early diagnosis of PDAC, it is important to clarify the frequency of FPA in controls without PDAC. We, therefore, analyzed pre-diagnostic CT performed at least 5 years before the diagnostic CT in 76 sex- and age-matched control subjects without PDAC. We used this time interval to minimize the likelihood of unidentifiable PDAC in the control subjects. The median age of the control subjects was 72 (range 42–95) years at the time of diagnostic CT and the time interval between the diagnostic CT and pre-diagnostic CT was 8.8 (range 5.01–20.6) years. FPA was observed in 3/76 (3.9%) control subjects on pre-diagnostic CT: two with hepatocellular carcinoma and one with gastric cancer. FPA was also observed on diagnostic CT in all of these control subjects. FPA was more frequently observed on pre-diagnostic CT in patients with PDAC than controls without PDAC (*p* < 0.001). FPA could predict the subsequent diagnosis of PDAC with a sensitivity of 34.2%, specificity of 96.1%, a positive predictive value of 89.7%, and a negative predictive value of 59.3%.

### 3.5. Case Presentation

**Figure 1:**
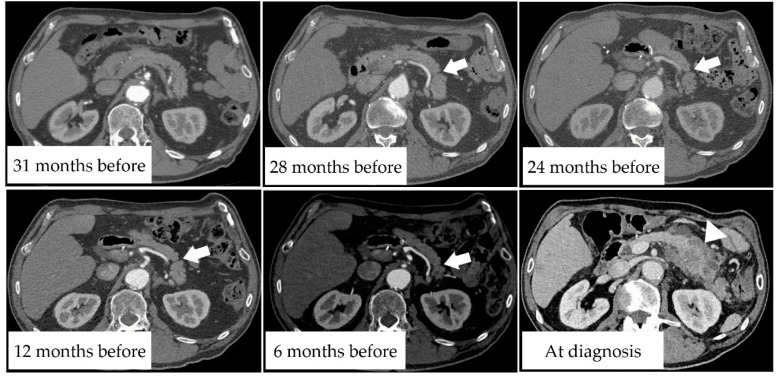
A man in his late 70 s with pancreatic tail ductal adenocarcinoma. Pre-diagnostic CT scans at 31, 28, 24, 12, and 6 months before PDAC diagnosis showing temporal changes in the pancreatic tail. White arrows indicate focal pancreatic atrophy, and the white arrowhead indicates invasive pancreatic ductal adenocarcinoma.

Here, we present two representative cases of FPA detected on pre-diagnostic CT. Case 1 was that of a man in his late 70 s who had undergone thoracic endovascular aortic repair for an aortic aneurysm five years before presentation and underwent regular CT examinations. Figure 1 shows the CT scans of the pancreas over time. The CT scan obtained 28 months before PDAC diagnosis shows slit-like FPA in the pancreatic tail (Figure 1; white arrow). On CT scans obtained at 24 months before PDAC diagnosis, FPA was more visible. On the CT scan taken at 12 months, MPD dilatation appeared. On the CT scan obtained at 6 months, MPD dilatation progressed and the pancreatic tail became atrophic. Subsequently, the patient became aware of his appetite loss and abdominal pain. CT revealed a tumor measuring 30 mm in diameter and he was diagnosed with stage IV PDAC (Figure 1; white arrowhead).

**Figure 2:**
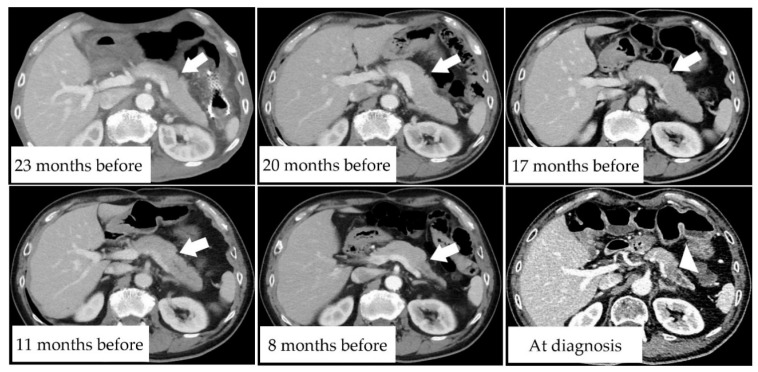
A man in his early 70s with pancreatic tail ductal adenocarcinoma (PDAC). Pre-diagnostic CT scans at 23, 20, 17, 11, and 8 months before PDAC diagnosis showing temporal changes in the pancreatic tail. White arrows indicate focal pancreatic atrophy, and the white arrowhead indicates invasive pancreatic ductal adenocarcinoma.

Case 2 was that of a man in his early 70 s who underwent radical surgery for colon cancer three years before presentation and underwent regular CT examination. Figure 2 shows the CT scans of the pancreas over time. The CT scan obtained at 23 months before PDAC diagnosis revealed a slit-like FPA in the pancreatic tail (Figure 2; white arrow). FPA was also observed on CT scans taken at 17 and 20 months. On the CT scan taken at 11 months, FPA became more visible, and MPD dilatation appeared. On the CT scan taken at 8 months, MPD dilatation became more visible, and the pancreatic tail became atrophic. Subsequently, diagnostic CT revealed a pancreatic mass measuring 21 mm in diameter (Figure 2; white arrowhead). The patient underwent a distal pancreatectomy and was pathologically diagnosed with stage IIB PDAC.

## 4. Discussion

**Figure 3:**
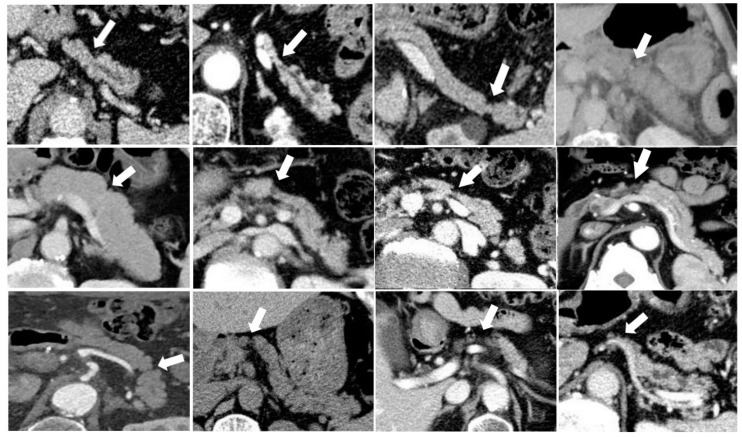
CT images of various types of focal parenchymal atrophy (white arrows).

In this case–control study, we showed that FPA, which has been reported as a characteristic imaging sign of early stage PDAC [13,14,15], was observed in about one-third of the patients at least 6 months before PDAC diagnosis (Figure 3). FPA was more frequently observed in patients with PDAC than sex- and age-matched controls without PDAC. Importantly, FPA was observed in the absence of tumor masses on CT in all the 26 patients, indicating that the clinical disease stage, if determined, was presumably Stage 0 or I at that time. If FPA was recognized properly as a sign suggesting early stage PDAC, these patients might have undergone further examinations for PDAC at that time and might have been diagnosed with PDAC at relatively earlier stages. Importantly, FPA could be detected on pre-diagnostic CT in patients undergoing unenhanced CT only. This is in contrast to other imaging findings, such as MPD changes, cysts, and tumors, which usually require enhanced CT [11].

FPA was less frequently observed in PDAC located in the pancreatic head. One explanation for this might be the difficulty in detecting FPA, if any, in the pancreatic head owing to its anatomical thickness, compared to the pancreatic body and tail [23]. Although the pancreatic head is the predominant location of PDAC, usually accounting for more than 50% of all cases [24], previous studies have shown a relatively low frequency of stage 0 PDAC in the pancreatic head, compared to that at other locations in the pancreas. In a multi-center study conducted in Japan, 17/51 (33.3%) stage 0 PDAC were located in the pancreatic head, and the remaining 34/51 (66.7%) ones were located in the pancreatic body and tail [16]. Nakahodo et al. [15] reported 27 cases of stage 0 PDAC, and only 5 (18.5%) PDAC were located in the pancreatic head. PDAC in the uncinate process, especially at PDAC at an early stage, can be overlooked on imaging owing to the absence of biliary or pancreatic ductal dilatation [11]. MPD dilation, which is another important clue for the early diagnosis of PDAC [11,12,13], is recognized more easily in the pancreatic body and tail than in the pancreatic head because MPD is intact in the pancreatic head [13]. Notably, Matsudo et al. [25] reported that carcinoma in situ was observed more frequently in the pancreatic body and tail than in the pancreatic head in autopsied cases, suggesting that the mechanism of carcinogenesis differs depending on the tumor location in the pancreas. Genetic and molecular features might differ between the pancreatic head and pancreatic body/tail PDAC [26]. In either case, our results support the notion that early diagnosis of PDAC in the pancreatic head is difficult.

As screening for PDAC is not recommended in individuals who are at average risk for PDAC [27], screening in those who are at high risk of PDAC, including those with new-onset diabetes, provides diagnostic opportunities [28,29]. In addition, follow-up/workup of other diseases provides diagnostic opportunities for PDAC in asymptomatic subjects. In this study, more than half of the patients underwent pre-diagnostic CT for follow-up of non-pancreatic diseases. In a multi-center study conducted in Japan, PDAC was detected due to abnormalities during the follow-up/workup of other diseases in approximately 50% of patients with early stage PDAC [16]. Patients with incidentally detected PDAC during examination for other diseases have a better prognosis than those with PDAC diagnosed on symptom presentation [29,30]. FPA should be monitored closely in subjects undergoing abdominal CT.

The underlying mechanisms that lead to the development of FPA remain unclear. Pathological examination of the resected pancreatic tissue has revealed that FPA is associated with the loss of acinar cells and the subsequent replacement by fat or fibrosis [13,14,15]. The stenosis of the peripheral branch duct due to the peripheral pancreatic intraepithelial neoplasia lesions might lead to FPA of the area of indicated branch duct; however, such findings were not evident in the resected specimens in our study (data not shown). The development of pancreatic intraepithelial neoplasia is associated with lobular atrophy of the surrounding pancreatic parenchyma in patients with a strong family history of PDAC [31]. The interactions between early stage cancer cells and the microenvironment, including adipocytes, might play a role in this phenomenon [32,33]. The cancer-promoting effects of adipocytes have been recently reported [34]. As the disease progresses, rapid tumor expansion and extensive distal atrophy occur, and FPA will no longer be recognized. It is unknown whether such interactions in the tumor microenvironment, if any, are limited to early stage cancer cells or applicable to PDAC cells. This is an important topic for future research because fat replacement might result from, and be a predisposing factor for, pancreatic carcinogenesis. Pancreatic fat infiltration is involved in the development of PDAC [35] and is associated with a higher risk of PDAC [36].

In our study, the time course of the presentation was different between MPD changes and FPA; the frequency of MPD cut-off/dilatation increased as the time of PDAC diagnosis approached, whereas the frequency of FPA was stable and FPA disappeared at the time of PDAC diagnosis in 21/26 (80.8%) patients. Even on CT performed at 2 and 3 years before PDAC diagnosis, 9/41 (22.0%) patients presented with FPA but none presented with MPD changes, suggesting that FPA presents earlier than MPD changes. Singh et al. [17] reported that changes on CT become visible between 12 and 18 months before PDAC diagnosis, with the MPD cut-off without a mass being the earliest sign; the presence or absence of FPA was not described in their study. Very recently, Toshima et al. [37] analyzed abnormalities of the pancreas on CT performed at least 1 year before the diagnosis of clinical stage I PDAC; they showed that a focal pancreatic abnormality was present on the most recent pre-diagnostic CT scan in 55/103 (53.4%) patients with PDAC and that the most common focal abnormality was atrophy, which accounted for 39/103 (37.9%) cases. The mean duration from initial appearance to PDAC diagnosis was 4.6 years for focal atrophy and 1.1 years for MPD changes, suggesting that FPA appears before MPD changes and retains for a longer period. The relatively long presentation of FPA might reflect its slow and prolonged progression during the precursor stage [38], providing a wide window for the early diagnosis of PDAC.

This study has several limitations, including its retrospective nature, a limited number of patients, varying intervals between CT, varied scanning protocol, and varying durations of the observation period. The possibility could not be completely denied that the control subjects would eventually develop PDAC in the future after long-term surveillance. Contrast-enhanced CT was not performed in all PDAC patients, and we might not have been able to detect MPD dilatation and pancreatic cysts on unenhanced CT in some cases. In many patients with PDAC presenting with FPA, CT scans were not available before the appearance of FPA, and the duration of FPA appearance was unclear. Nevertheless, our study suggests that FPA predicts the subsequent diagnosis of PDAC and is useful for the early diagnosis of PDAC. Although we enrolled the patients with PDAC diagnosed at our institute, pre-diagnostic CT was performed in 26 institutions including ours. FPA was observed on pre-diagnostic CT performed at other institutes, as well as ours. Although the sensitivity of subsequent PDAC prediction was 34.2% and not so high, CT examination was not performed for the diagnosis of PDAC but for follow-up of non-pancreatic diseases. Importantly, the specificity of FPA for PDAC diagnosis was as high as 96.1% but not 100%; FPA is sometimes detected in healthy subjects and those with benign diseases, ranging from 7.8% to 15.8% [13,14,15]. Indeed, FPA was observed in 3.9% of control subjects without PDAC in this study. It is reasonable to assume that further examinations for PDAC, including magnetic resonance cholangiopancreatography, endoscopic ultrasonography, and occasionally endoscopic retrograde cholangiopancreatography, should be performed if FPA is first detected in subjects at a high risk of PDAC, including subjects from families with familial pancreatic cancer [39] or subjects with other findings suggestive of PDAC, such as MPD changes. If PDAC is not diagnosed at that time, careful follow-up of patients would be required. As all previous studies were retrospective, the proper follow-up protocol, including the interval of examinations and imaging modalities, is unknown. Further studies, including multi-center prospective studies, are warranted to clarify these issues.

## 5. Conclusions

In this study, we showed that FPA was present on pre-diagnostic CT scans obtained between 6 months and 3 years before PDAC diagnosis in about one-third of patients with PDAC, and FPA was more frequently found in patients with PDAC than in those without PDAC. FPA might be the earliest sign predicting the subsequent diagnosis of PDAC that appears before MPD changes and mass identification. We recommend close monitoring of FPA in surveillance programs for high-risk populations and in abdominal CT performed for other indications. Further recognition of FPA as an important predictor of subsequent PDAC development would lead to early diagnosis and consequent improvements in the prognosis of this intractable disease.

## Figures and Tables

**Table 1:** Characteristics of the patients who underwent pre-diagnostic CT.

	PDAC (*N =* 76)
Male, N (%)	43 (56.6)
Age at PDAC Diagnosis, Median (Range)	72 (49–85)
Location of PDAC, N (%)	
** **Head	27 (35.5)
** **Body	30 (39.5)
** **Tail	19 (25.0)
Pancreatic resection, N (%)	45 (59.2)
Mass diameter, mm, median (range)	21.5 (0–80.0)
UICC Stage, N (%)	
** **Stage 0	1 (1.3)
** **Stage IA	15 (19.7)
** **Stage IB	10 (13.2)
** **Stage IIA	4 (5.3)
** **Stage IIB	16 (21.1)
** **Stage III	8 (10.5)
** **Stage IV	22 (28.9)
Reason for pre-diagnostic CT, N (%)	
** **Follow-up for pancreatic disease	32 (42.1)
** **Follow-up for various cancers	27 (35.5)
** **Follow-up for liver disease	4 (5.3)
** **Follow-up for cardiovascular disease	3 (3.9)
** **Follow-up for gastrointestinal disease	2 (2.6)
** **Follow-up for respiratory disease	2 (2.6)
** **Other	6 (7.9)

CT, computed tomography; PDAC, pancreatic ductal adenocarcinoma; UICC, Union for International Cancer Control.

**Table 2:** CT findings by time interval before the diagnosis of PDAC.

	At Diagnosis (*N =* 76)	6 Months–1 Year before (*N =* 44)	1–2 Years before (*N =* 51)	2–3 Years before (*N =* 41)
Time interval before the diagnosis, years, median (range)	-	0.64(0.50–0.99)	1.27 (1.00–1.96)	2.20 (2.00–2.99)
Contrast-medium use, N (%)	76 (100)	35 (79.5)	42 (82.4)	33 (80.5)
Mass, N (%)	63 (82.9)	10 (22.7)	0 (0)	0 (0)
Mass diameter, mm, median (range)	21.5 (0–80.0)	17.5 (11.0–26.0)	0 (0)	0 (0)
MPD cut-off/dilatation, N (%)	58 (76.3)	12 (27.3)	7 (13.7)	1 (2.4)
Pancreatic cyst within 2 cm of tumor, N (%)	22 (28.9)	12 (27.3)	18 (35.3)	12 (29.3)
Pancreatic cyst beyond 2 cm of tumor, N (%)	28 (36.8)	14 (31.8)	13 (25.5)	11 (26.8)
Focal parenchymal atrophy, N (%)	6 (7.9)	14 (31.8)	14 (27.5)	9 (22.0)
Location of PDAC, N (%)				
** **Head	27 (35.5)	18 (40.9)	18 (35.3)	14 (34.1)
** **Body	30 (39.5)	15 (34.1)	18 (35.3)	15 (36.6)
** **Tail	19 (25.0)	11 (25.0)	15 (29.4)	12 (29.3)
UICC Stage at diagnosis, N (%)				
** **Stage 0	1 (1.3)	1 (2.3)	0 (0)	0 (0)
** **Stage IA	15 (19.7)	6 (13.6)	13 (25.5)	9 (22.0)
** **Stage IB	10 (13.2)	4 (9.1)	7 (13.7)	5 (12.2)
** **Stage IIA	4 (5.3)	2 (4.5)	2 (3.9)	2 (4.9)
** **Stage IIB	16 (21.1)	13 (29.5)	7 (13.7)	8 (19.5)
** **Stage III	8 (10.5)	6 (13.6)	6 (11.8)	5 (12.2)
** **Stage IV	22 (28.9)	12 (27.3)	16 (31.4)	12 (29.3)

CT, computed tomography; MPD, main pancreatic duct; PDAC, pancreatic ductal adenocarcinoma; UICC, Union for International Cancer Control.

**Table 3:** Characteristics of the patients presenting focal parenchymal atrophy.

	At Diagnosis (*N =* 6)	6 Months–1 Year before (*N =* 14)	1–2 Years before (*N =* 14)	2–3 Years before (*N =* 9)
Time interval before the diagnosis, years, median (range)	-	0.62 (0.50–0.99)	1.33 (1.00–1.70)	2.61 (2.05–2.99)
Contrast-medium use, N (%)	6 (100)	12 (85.7)	10 (71.4)	8 (88.9)
Mass, N (%)	0 (0)	0 (0)	0 (0)	0 (0)
MPD cut-off/dilatation, N (%)	5 (83.3)	5 (35.7)	1 (7.1)	0 (0)
Pancreatic cyst within 2 cm of tumor, N (%)	1 (16.7)	4 (28.6)	5 (35.7)	2 (22.2)
Pancreatic cyst beyond 2 cm of tumor, N (%)	2 (33.3)	2 (14.3)	4 (28.5)	4 (44.4)
Location of PDAC, N (%)				
** **Head	1 (16.7)	1 (7.1)	2 (14.2)	1 (11.1)
** **Body	4 (66.7)	8 (57.1)	8 (57.1)	5 (55.5)
** **Tail	1 (16.7)	5 (35.7)	4 (28.6)	3 (33.3)
UICC Stage at diagnosis, N (%)				
** **Stage 0	1 (16.7)	1 (7.1)	0 (0)	0 (0)
** **Stage IA	4 (66.6)	1 (7.1)	4 (28.6)	1 (11.1)
** **Stage IB	0 (0)	2 (14.3)	3 (21.4)	3 (33.3)
** **Stage IIA	0 (0)	0 (0)	0 (0)	1 (11.1)
** **Stage IIB	1 (16.7)	3 (21.4)	2 (14.3)	1 (11.1)
** **Stage III	0 (0)	4 (28.6)	1 (7.1)	1 (11.1)
** **Stage IV	0 (0)	3 (21.4)	4 (28.6)	2 (22.2)

MPD, main pancreatic duct; PDAC, pancreatic ductal adenocarcinoma; UICC, Union for International Cancer Control.

**Table 4:** Comparison of the cases presenting with FPA and those who did not on pre-diagnostic CT.

	With FPA (*N =* 26)	Without FPA (*N =* 50)	*p* Value
Number of pre-diagnostic CT, N (%)			0.50
1	13 (50.0)	21 (42.0)	
2	9 (34.6)	15 (30.0)	
3	4 (15.4)	14 (28.0)	
Male, N (%)	13 (50.0)	30 (60.0)	0.47
Age at PDAC diagnosis, median (range)	73 (65-80)	71 (49-85)	0.38
Mass, N (%)	21 (80.8)	42 (84.0)	0.75
Mass diameter, mm, median (range)	22.5 (0-80.0)	19.5 (0-66.0)	0.53
MPD cut-off/dilatation, N (%)	22 (84.6)	36 (72.0)	0.27
Pancreatic cyst within 2cm of tumor, N (%)	6 (23.1)	16 (32.0)	0.59
Pancreatic cyst beyond 2 cm of tumor, N (%)	9 (34.6)	19 (38.0)	0.80
Location of PDAC, N (%)			0.007
Head	3 (11.5)	24 (48.0)	
Body	14 (53.8)	16 (32.0)	
Tail	9 (34.6)	10 (20.0)	
UICC Stage at diagnosis, N (%)			0.81
Stage 0	1 (3.8)	0 (0)	
Stage IA	4 (15.4)	11 (22.0)	
Stage IB	4 (15.4)	6 (12.0)	
Stage IIA	1 (3.8)	3 (6.0)	
Stage IIB	5 (19.2)	11 (22.0)	
Stage III	4 (15.4)	4 (8.0)	
Stage IV	7 (26.9)	15 (30.0)	

CT, computed tomography; FPA, focal parenchymal atrophy; MPD, main pancreatic duct; PDAC, pancreatic ductal adenocarcinoma; UICC, Union for International Cancer Control.
